# Active Probing of a RuO_2_/CZ Catalyst Surface as a Tool for Bridging the Gap Between CO Oxidation Catalytic Tests in a Model and Realistic Exhaust Gas Stream

**DOI:** 10.1021/acsmaterialsau.4c00062

**Published:** 2024-09-24

**Authors:** Ewa M. Iwanek (nee Wilczkowska), Leonarda Francesca Liotta, Giuseppe Pantaleo, Linje Hu, Shazam Williams, Donald. W. Kirk, Zbigniew Kaszkur

**Affiliations:** † Faculty of Chemistry, Warsaw University of Technology, Noakowskiego 3, 00-664 Warsaw, Poland; ‡ Istituto per lo Studio di Materiali Nanostrutturati (ISMN)-CNR, Palermo I-90146, Italy; § DCL International Inc., Concord, Ontario L4K 4T5, Canada; ∥ Department of Chemical Engineering and Applied Chemistry, University of Toronto, 200 College St., Toronto, Ontario M5S3E5, Canada; ⊥ Institute of Physical Chemistry, Polish Academy of Sciences, Kasprzaka 44/52, 01-224 Warsaw, Poland

**Keywords:** heterogeneous catalyst, solid solution support, carbon monoxide oxidation, ruthenium dioxide, exhaust cleanup

## Abstract

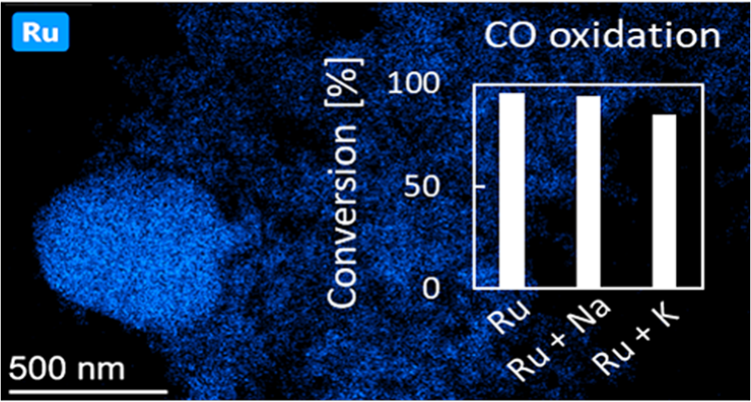

Herein, we present a paper that attempts to bridge the gap between CO oxidation catalytic tests performed in a model stream and a more realistic exhaust gas stream by incorporating characterization methods that allow for active probing of the catalyst surface. The results have shown that it is not just the abundance of a given type of species on the surface that impacts the activity of a system but also the ease of extraction of ions from their surface (time-of-flight secondary ion mass spectrometry) and the response of the support to change in the feed composition (dynamic in situ X-ray diffraction (XRD) with variable atmosphere). The study utilizes the method of doping a catalyst (RuO_2_/CZ) with a small amount of alkali-metal (K^+^ or Na^+^) carbonates in order to slightly modify its surface to gain insight into parameters that may cause discrepancies between model stream activity and complex stream activity. The most pronounced difference is that in the model stream, which contains only CO and O_2_ in helium, both alkali ions improve the activity of the system at temperatures around 175 °C, whereas in the complex stream, which mimics the exhaust stream from a diesel engine under oxygen lean conditions, the K^+^-doped catalyst is slightly worse than RuO_2_ /CZ and RuO_2_ + Na^+^/CZ and much worse in propane combustion. The total hydrogen consumption values (temperature-programmed reduction) and the O_ads_/O_latt_ ratios (X-ray photoelectron spectroscopy) both place the RuO_2_ + K^+^/CZ system between the other two and hence provided no reason for the unusual behavior of the K^+^-doped catalyst. In contrast, both in situ XRD measurement tests and ToF SIMS results show a pronounced difference between the RuO_2_ + K^+^/CZ catalyst and the other two systems, which indicates that the interaction of the surfaces with the reagents might be the cause of the discrepancy. The CO_2_-TPD results show that this system retains more CO_2_, i.e., the product, at adsorption sites, which might reduce the adsorption of other reagents, i.e., oxygen ions, CO, and propane, hence lowering the overall activity of the system.

## Introduction

Restrictions on the emission from mobile engines have become increasingly more stringent, e.g., in the European Union, a series of directives have been issued: Euro1–Euro7, which are amendments to Directive 70/220/EEC^1^ and provide standards for exhaust emission for different vehicle categories. In the case of carbon monoxide emissions, they have been reduced gradually in the consecutive directives. Hence, continuous work on the improvement of the active phase of catalytic converters, as well as the monoliths themselves is needed.^2^ Studies aimed at replacing the ceramic monoliths in mobile engines by metallic ones have shown numerous advantages of the metallic substrate,^3^ such as (1) larger geometric surface area, which enables higher flows and a lower pressure drop, (2) lower thermal mass (heat capacity), and (3) higher thermal and mechanical durability. However, the metallic substrate has thus far only been implemented in stationary engines (power plants, turbines, etc.) due to its higher cost, which limits its use for vehicle applications. In the case of either monolith, it is the active phase that determines the overall activity of the converter. The active phase of the converter has to meet the CO emission standards, which is especially difficult under oxygen lean conditions. Zirconium-doped ceria^4−6^ has been frequently studied and applied in CO oxidation as either the active components or the support, though numerous new CO oxidation catalysts continue to be investigated.^7−9^ Multiple components of the real exhaust stream render some of the results obtained in model streams untransferable. Hence, in this study, the same catalyst is tested in both a model stream and a complex stream.

CO oxidation is one of the reactions for which ruthenium-based catalysts have been used.^10−15^ Although ruthenium-based systems are often studied in the form of organometallic complexes in homogeneous catalysis and are used in metathesis reactions,^16−18^ in electrochemistry^19,20^ and in photocatalysis,^21^ they have also been applied as a heterogeneous ruthenium catalyst in CO oxidation,^10−15^ as well as CO methanation,^22^ CO_2_ methanation,^23,24^ catalytic oxidation of ammonia,^25^ and ammonia synthesis.^26,27^ Out of these reactions, the most studied reaction is CO oxidation. Several studies were performed with Ru particles supported on ceria,^28−30^ but none use ruthenium oxide deposited on Ce_1–*x*_Zr_*x*_O_2_. Titania and carbon-supported Ru systems have been studied in great detail, but the interest in CeO_2_-supported systems has been gaining momentum due to the substantial interaction between ruthenium and ceria, as indicated in studies regarding CO oxidation^30^ and CO_2_ methanation.^29^ In both cases, enhanced activity is associated with oxygen vacancies. This in turn is known to also be affected by the incorporation of zirconium ions into the ceria lattice.^31^ Since zirconium-doped ceria is commonly used in automotive exhaust cleanup catalysts, this solid solution should also be investigated.

In the case of carbon monoxide oxidation, the core–shell structure with a metallic ruthenium center covered with a thin layer of RuO_2_ has been the topic of studies^32^ and is believed to be the most active form of a ruthenium catalyst. However, some other groups reported ruthenium oxide as the active phase^30,33−35^ rather than a core–shell type of Ru–RuO_2_ system, which is why no steps were taken to reduce RuO_2_ in this study. The mechanism of CO oxidation on ruthenium catalysts has been postulated to take place on coordinatively unsaturated sites (*cus* Ru) and involves strongly bonding bridging CO molecules, which predominantly react with on-top O, whose activation barrier is much lower than that of bridging O (O_br_).^10^ In contrast to gold nanoparticles, the activity of nanoruthenium particles in CO oxidation has been shown to increase upon nanoparticle size increase.^36^ The addition of alkali-metal ions would not likely be implemented in exhaust cleanup catalysts nor is it meant to improve the activity but to serve as a useful tool to slightly modify the surface of the catalyst without changing important parameters such as support and active phase particle size, shape, distribution, etc. to gain insight into what makes it active, as in the case of the previously studied gold catalyst.^37^ The literature contains very little information regarding which parameters associated with surface oxygen determine the activity of ruthenium catalysts in this reaction. This paper aims to fill that gap by actively probing the surface oxygen concentration and availability, as well as the ease/form in which oxygen can be extracted, and other properties that can be relevant for this particular reaction.

## Experimental Section

### Materials

The following chemicals were used without further purification to make the catalysts: zirconyl nitrate (reagent grade, POCh Gliwice), ammonium ceric nitrate (reagent grade, POCh Gliwice), RuCl_3_·*x*H_2_O (reagent grade, Sigma-Aldrich), K_2_CO_3_ anhydrous (reagent grade, POCh Gliwice), Na_2_CO_3_ anhydrous (reagent grade, POCh Gliwice), and aqueous ammonia solution (25%, POCH Gliwice).

### Catalyst Preparation

The support was prepared in accordance with the procedure described by us earlier.^37^ In brief, the appropriate amounts (to obtain a final Ce/Zr ratio of 85:15) of each precursor (zirconyl nitrate and ammonium ceric nitrate) were dissolved in 500 and 100 mL of redistilled water, respectively. The solutions were added, and the mixtures were manually stirred for 5 min. The mixture was then transferred into a beaker equipped with a stir bar and placed on a magnetic stirrer, and the hydroxides were precipitated using an excess of an aqueous ammonia solution. The obtained product was thoroughly washed with redistilled water and dried at 90 °C overnight. The support (denoted as CZ) was calcined for 4 h at 550 °C in a muffle furnace. Next, the support was placed in a beaker with 500 mL of redistilled water. An appropriate amount of ruthenium chloride (based on the assumptions that *x* in RuCl_3_·*x*H_2_O equals 2 and a deposition efficiency of 90%) was dissolved in 100 mL of redistilled water and added to the beaker, and a 30% solution of Na_2_CO_3_ was added to adjust the pH to approximately 8. The mixture was stirred for 6 h, allowed to age for 1 day, then transferred into a Buchner funnel, and washed with 3 L of redistilled water to remove the sodium ions from the precipitate and obtain a neutral pH of the filtrate. The solid was dried at 90 °C for 1 h and calcined for 1 h at 550 °C to obtain the catalyst.

Next, the ruthenium catalyst was characterized and divided into three portions, two of which were then mixed with redistilled water and the appropriate amount of carbonates (1 mL of a solution prepared using 0.024 and 0.032 g of Na_2_CO_3_ and K_2_CO_3_, respectively, dissolved in 50 mL of redistilled water was used per 0.2 g of catalyst) to obtain a loading of 0.3 at. % of each alkali ion on the surface.

### Catalytic Activity

#### CO Oxidation in the Model Stream

The experiments were performed in a tubular flow-through quartz reactor containing 0.05 g of the catalyst with the gas mixture flowing at 50 mL/min in the temperature range of 30–300 °C. The model stream consisted only of 1% CO, 1% O_2_, and balance helium. The composition of the outlet stream was monitored by using an infrared elemental analyzer.

#### CO Oxidation in the Complex Stream

For comparison with the model stream, the same catalysts were tested in the complex stream, which simulates the diesel engine exhaust stream under lean oxygen conditions. The experiments were carried out in a stainless steel, flow-through reactor lined with a quartz tube. A stream consisting of CO (1000 ppm), 10% O_2_, and hydrocarbons (1000 ppm of CH_4_, 150 ppm of C_2_H_6_, and 50 ppm of C_3_H_8_), as well as 200 ppm of NO, 7% water vapor, 5% CO_2_, and balance N_2_ was passed through the fixed catalyst bed (approximately 0.5 g) at a rate of 1.5 L/min. The post reaction mixture was passed through an MKS MultiGas 2030 FTIR analyzer to determine its composition. Due to the presence of water vapor, the experiments were conducted in the temperature range of 115–550 °C.

### Characterization

Ex situ powder X-ray diffraction experiments were conducted in the scattering angle range of 15–150°, with 0.02° step size. A D5000 diffractometer from Bruker (AXS GmbH) with a LynxEye detector was used to obtain the diffraction patterns. A sealed tube copper anode (Kα radiation λ = 1.5418 Å) was operated at 40 kV, 40 mA. Bragg–Brentano divergent beam optics (Kβ filter) were applied. The background was subtracted, and the peaks were fitted with Fityk v.1.3.0 software. In situ X-ray diffraction (XRD) tests were carried out in the following sequence: (1) He at room temperature, (2) CO (5%, balance He) stream at 200 °C, (3) He stream at 200 °C, (4) O_2_ stream (5%, balance He) at 200 °C, (5) CO (8%), O_2_ (4%), balance He at 200 °C, (6) He stream at 200 °C, (7) reduction in a H_2_ stream at 400 °C, 20% in He, and (8) room-temperature measurement in He. The gases used were CO (99.998%, Linde), O_2_ (5N, Multax), H_2_ (5N, Multax), and He (5N, Multax). Before the measurements, the catalyst was kept in a stream of He for 2 h.

High-resolution transmission electron microscopy (HRTEM) imaging was performed with a TALOS F200X transmission electron microscope (Thermo Fisher Scientific) with an X-ray Field Emission Gun and an electron beam energy of 200 keV. The instrument is equipped with four in-column Silicon Drift Detectors, which have a 120 mm^2^ active area. Energy dispersive X-ray spectroscopy mapping was performed using high-angle annular dark-field (HAADF) in scanning TEM (STEM) mode. Prior to analysis, the catalysts were suspended in 2 cm^3^ of ethanol using a sonic bath, and the suspension was deposited onto a copper grid.

Time-of-flight secondary ion mass spectrometry experiments were performed using a Helios 5 Dual Beam microscope from Thermo Fisher Scientific, equipped with a ToF SIMS detector and ToF Werk software. Prior to measurements, the eucentric position was set using the electron beam operating with an Everhart–Thornley Detector and the Through Lens Detector for the ion beam. The analysis was performed in both positive and negative ions modes. The parameters used for the measurements were as follows: beam current: 4 nA; beam voltage: 30 kV; resolution: 512 × 442; horizontal field width: 50 μm; number of frames: 300; dwell time: 10 μs; with group delay disabled. The hydrogen peak was used for the calibration.

The chemical environment of the species was investigated by using X-ray photoelectron spectroscopy (KAlpha, ThermoScientific). The measurements were performed by using Al Kα (*h*ν = 1486.6 eV) radiation. Survey spectra and detailed regions were recorded using a 250 μm analysis size and were recorded in the constant analyzer energy (CAE) mode: CAE 200 eV, 3 scans, 1.0 eV step and CAE 50 eV, 15 scans, 0.1 eV step, respectively. All peaks except the Ru 3d doublet components were fitted using Avantage software (ThermoScientific) with symmetrical Lorentzian–Gaussian-type curves with a 30:70 ratio after background subtraction and BE shift to O 1s to 530.0 eV. The Ru 3d + C 1s region was fitted as described by Morgan^38^ and Rochelfort et al.^39^

Temperature-programmed reduction (H_2_-TPR) and temperature-programmed desorption of carbon dioxide (CO_2_-TPD) experiments were conducted using Autochem 2910 from Micromeritics equipped with a thermal conductivity detector (TCD) with samples of approximately 100 and 300 mg, respectively. During TPR, the sample was heated to 150 °C (10 °C/min) in a flow of helium containing 5 vol % O_2_ (30 mL/min) in order to clean the surface, then, after 30 min, it was cooled in flowing He (30 mL/min). Next, a mixture of argon and 5 vol % H_2_ (30 mL/min) was used for the reduction as the sample was heated to 1050 °C at a rate of 10 °C/min. The amount of hydrogen consumed was determined by integration of the curve peaks, applying a calibration curve registered using H_2_ concentrations in the range of 0.5–5 vol % H_2_/Ar. The procedure of CO_2_-TPD experiments was as follows: (1) heating to 500 °C at a rate of 30 °C/min, (2) flushing with He at 500 °C flowing at 40 mL/min for 1 h; (3) cooling to 40 °C at 30 °C/min, (4) adsorption of CO_2_ at 40 °C in a flowing mixture of He/CO_2_ = 9:1 flowing at 40 mL/min for 2 h; (5) flushing with He 40 °C flowing at 40 mL/min, 1 h, and (6) temperature-programmed desorption of CO_2_ was performed in He while heating the sample to 800 °C at a rate of 10 °C/min. The area of the peaks was integrated and quantified by using a calibration curve.

Simultaneous thermal analysis coupled with mass spectrometry was used to monitor the mass loss steps and the thermal effects associated with them during the calcination of the catalyst after alkali carbonate deposition. Differential thermal analysis–thermogravimetry–mass spectrometry (DTA-TG-MS) tests were performed with a temperature ramp of 15 °C/min (the same rate as that in the muffle furnace) up to 550 °C in a flow of synthetic air (5N, PRAXAIR) at 90 mL/min. DTA-TG-MS measurements were also used to gain further insight into the reduction of the catalysts. In these measurements, the temperature increased to 550 °C at a rate of 10 °C/min (the same rate as during the TPR experiments) in a stream consisting of 10% H_2_ and 90% Ar (90 mL/min). In both cases, the MS *m*/*z* = 18 (H_2_O) and *m*/*z* = 44 (CO_2_) signals were monitored. DTA-TGA-MS catalytic transfer hydrogenation tests were performed using 75 mg of the catalyst and 80 μL of the reaction mixture with a heating ramp of either 5 or 2 K/min with a Scan Bargraph mode of the mass spectrometer in the range of 0–300.

The specific surface area (SSA) of the samples was measured with N_2_ physisorption at −196 °C on ASAP 2020 equipment (Micromeritics). Prior to measurements, the samples (∼100 mg) were degassed at 150 °C for 2 h. The specific surface area was calculated using the Brunauer–Emmet–Teller (BET) equation in the standard pressure range of 0.05–0.3 p/p_0_. The ruthenium content of the catalysts was determined using X-ray fluorescence (XRF) spectrometry (MiniPal 4, PANalitical B.V.). The energy dispersive spectrometer operated with a 9 W Rh tube charged with up to 30 kV voltage and a semiconductor silicon drift detector (SDD). The morphology of the samples was observed using scanning electron microscopy (Prisma E, Thermo Fisher Scientific). The imaging was performed using the following parameters: 10 mm working distance, voltage of 5 kV with a spot size of 3. The elemental maps were acquired at the same working distance, a voltage of 15 kV, and spot size of 7.

## Results and Discussion

**Figure 1:**
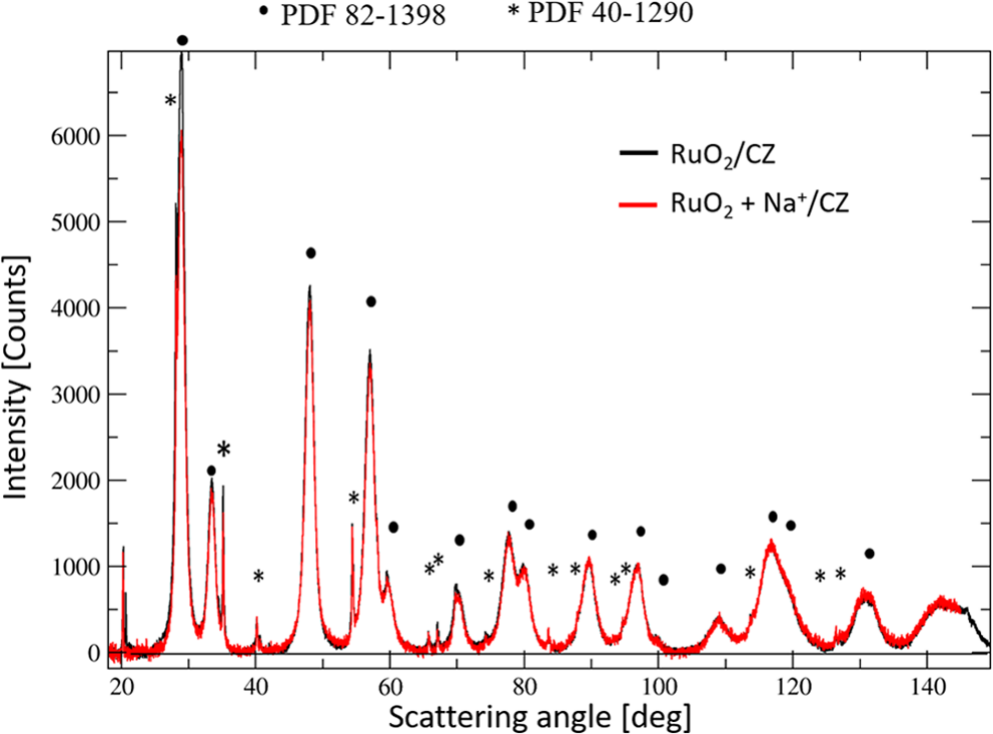
X-ray diffraction results obtained for RuO_2_/Ce_0.85_Zr_0.15_O_2_ prior to doping (black) and after doping with sodium carbonate (red).

Ruthenium hydroxide was deposited onto a large batch of the support using the precipitation–deposition method and was calcined before being divided into parts onto which alkali carbonate was subsequently deposited. This was done in order to avoid differences in the support composition (85 atom % Ce and 15 atom % Zr), as well as ruthenium loading (1.8 wt % as determined by XRF) and distribution. The X-ray diffraction pattern of the catalyst prior to alkali-metal ion deposition is shown in Figure 1. There are only two phases whose reflexes are visible in the diffraction pattern: ruthenium oxide (PDF 40-1290) and the single-phase solid solution support. The support exhibits the fluorite-type structure of ceria (PDF 82-1398) with zirconium ions present in the cerium sites. The radius of the zirconium ion is slightly smaller than that of Ce^4+^ (0.085 nm vs 0.101 nm), which leads to a slightly smaller lattice parameter than that of undoped ceria: 0.535 vs 0.541 nm. The doping of the catalyst with only 0.5 wt % of sodium does not lead to the presence of new reflections in the diffraction pattern, as seen in Figure 1. Similarly, in the XRF spectra both sodium and potassium were below the detection limit. Only the two most surface-sensitive techniques (energy dispersive X-ray spectroscopy, EDX, and X-ray photoelectron spectroscopy, XPS) have shown the presence of alkali-metal ions.

Since reflexes of the support overlap in the two diffraction patterns, it can be seen that the lattice parameter is not impacted by the doping. The same is true for the potassium ion-doped sample. No influence of doping is also seen in the surface areas of the three samples (Table 1), with a value close to 84 m^2^/g being noted for all three catalysts. The N_2_ adsorption/desorption isotherms and the hysteresis loop (Type IV) are the same for all of them; an example is provided in Figure S1. The rest of the values in Table 1, i.e., from TPR curves, XPS curves, and CO_2_-TPD curves, are discussed in the text alongside the figures with results of each of those techniques.

**Table 1:** Physicochemical Properties of Catalysts

sample parameter		RuO_2_/CZ	RuO_2_ + Na^+^/CZ	RuO_2_ + K^+^/CZ
SSA [m^2^/g]		85.3	84.1	83.8
TPR	*T*_max1_ [°C]/H_2_ consumption [cm^3^/g]	77/1.4	80/1.4	78/2.0
	*T*_max2_ [°C]/H_2_ consumption [cm^3^/g]	117/7.0	135/4.1	128/6.0
	*T*_max3_ [°C]/H_2_ consumption [cm^3^/g]	215/6.0	227/3.5	217/4.5
XPS	O_lattice_ (529.6 eV)	68	78	72
	O_ads_ (531.3 eV)	32	22	28
	100·Zr/(Ce + Zr)	32.2	31.5	37.0
	Ru [atom %]	6.07	3.79	4.85
TPD	CO_2_ desorbed [cm^3^/g]	3.17	3.97	4.33

**Figure 2:**
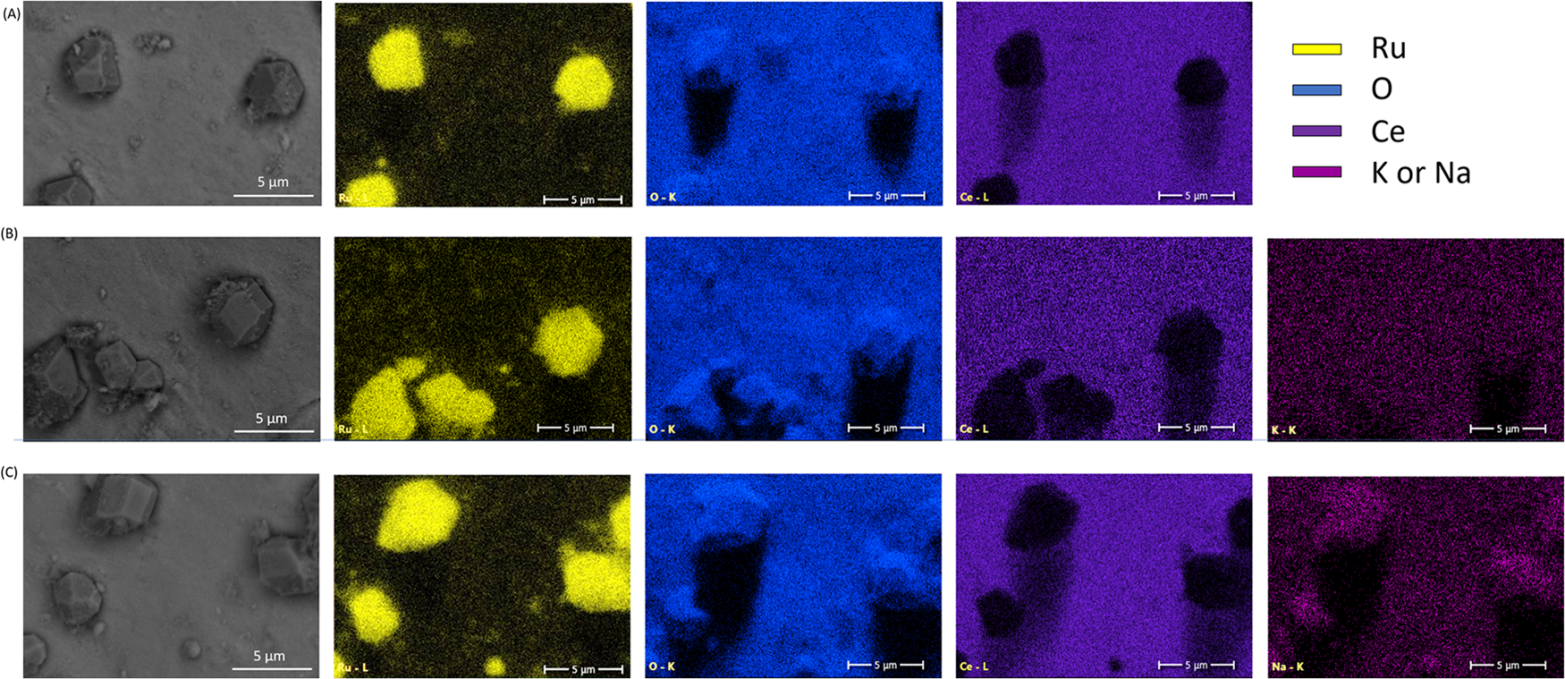
SEM-EDX results of the three catalytic systems: (A) RuO_2_/CZ, (B) RuO_2_ + K^+^/CZ, and (C) RuO_2_ + Na^+^/CZ.

Ruthenium oxide is present in the form of large, well-formed particles, as seen in the SEM image and elemental maps visible in Figure 2. The particles have well-developed facets and range in size, up to micrometer agglomerates visible in the elemental maps: indicated by yellow spots that are enriched in Ru with blue spots, which correspond to oxygen, in the same positions on top of the support with a lack of cerium in those spots. Similar particles are observed in the elemental maps obtained for the alkali ion-doped systems (Figure 2B,C)

**Figure 3:**
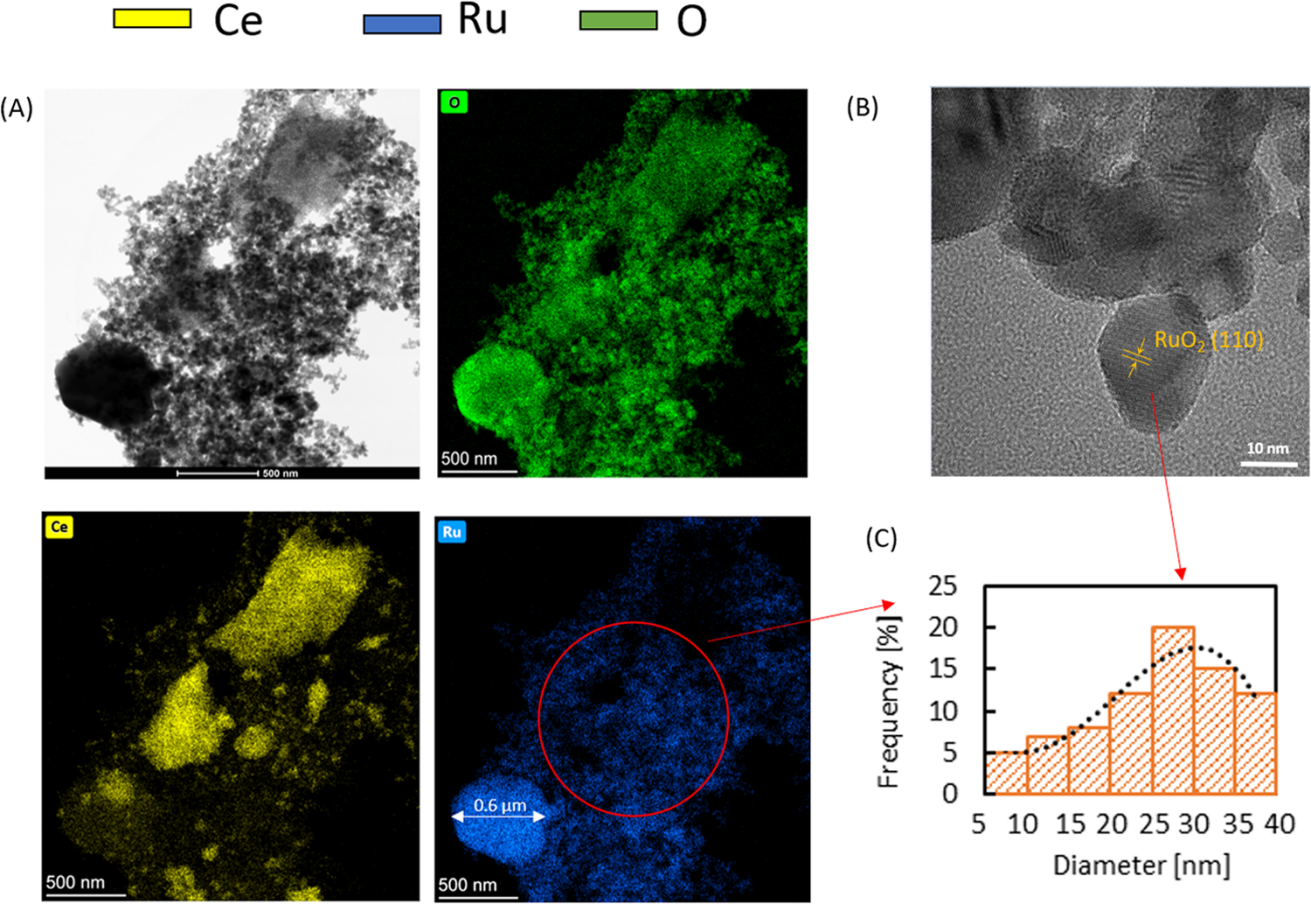
TEM results: (A) elemental distribution maps with (B) TEM image of RuO_2_/Ce_0.85_Zr_0.15_O_2_ (RuO_2_/CZ) particles at a magnification of 200 000 times and (C) histogram of nanometric RuO_2_ particles.

HRTEM investigation (Figure 3) into the particle sizes of ruthenium oxide indicates that apart from the large agglomerates as seen in the SEM-EDX images (Figure 2), there are much smaller, nanometric-sized particles, which means that there is a bimodal distribution of the active phase on the support: Figure 3C depicts the histogram with the distribution of only the nanometric particles, whereas the SEM images showed only the micrometric particles. One of the large ruthenium oxide particles (approximately 0.6 μm) is visible in the distribution map of ruthenium (blue), as well as a lot of small ones. Oxygen (green) is present in the same spots as cerium (yellow) and ruthenium.

The unfitted XPS results are all of the same, except for the detailed regions of the O 1s region (Figure S2). The comparison of the shapes of the signals indicates that the introduction of alkali-metal ions onto the surface of a support preloaded with ruthenium oxide does not change the chemical environment of ruthenium (Figure S2A; the binding energy values of the Ru 3p_3/2_ peaks are the same within 0.1 eV), the cerium ions (Figure S2B; the binding energy values of the *u*‴ peak are the same within 0.1 eV; the Ce^3+^/Ce^4+^ ratio is the same within 1–2%), or the zirconium ions (Figure S2C; the Zr 3d_5/2_ binding energy values are the same within 0.1 eV). The additional peak found in the Ru 3p region of the sodium-containing sample spectrum at the binding energy of 497–498 eV is due to a strong Auger KLL peak for Na; hence, it is not present in the spectra of the two other samples. Therefore, it can be stated that qualitatively, the chemical environment of ruthenium, cerium, and zirconium is the same in all three samples. This is logical considering the fact that all came from the same batch of RuO_2_/CZ, which after calcination was doped with an alkali-metal carbonate. Therefore, any differences in the surface composition can be attributed solely to deposition of the alkali-metal ions.

In all three cases, the main O 1s peak, which comes from lattice oxygen (530.0 eV), has a low-binding energy shoulder (531.7 eV) that corresponds to the concentration of the adsorbed oxygen (Figure S2E) but with a different O_ads_/O_lattice_ ratio. The values are given in Table 1. The ratio decreases in the order RuO_2_/CZ > RuO_2_ + K^+^/ CZ > RuO_2_ + Na^+^/CZ. Another difference is the noticeably higher Zr/(Ce+Zr) ratio noted for the Ru + Na sample (Table 1), i.e., 37% as compared to approximately 32% for both RuO_2_/CZ and RuO_2_ + K^+^/CZ. It is noteworthy that all three samples exhibit surface zirconium enrichment, i.e., about twice that of the bulk value (Table 1). The value for Na/CZ was 31.7%, which indicates that it is not a result of the interaction of sodium ions with the support but rather the joint interaction of RuO_2_ and Na^+^ with the support. The surface ruthenium content is also much higher than the average value but is larger for the undoped sample than for the alkali-doped systems (Table 1). Moreover, there is a clear change of the amount of the adsorbed oxygen in the top surface layer of the catalyst upon the addition of K^+^ and Na^+^, as evidenced by the differences in the O_latt_ and O_ads_ relative values in the O 1s region (Table 1 and Figure S2E). The addition of the alkali-metal ions led to a decrease in the amount of adsorbed oxygen, with a larger difference observed for the sodium-containing sample.

**Figure 4:**
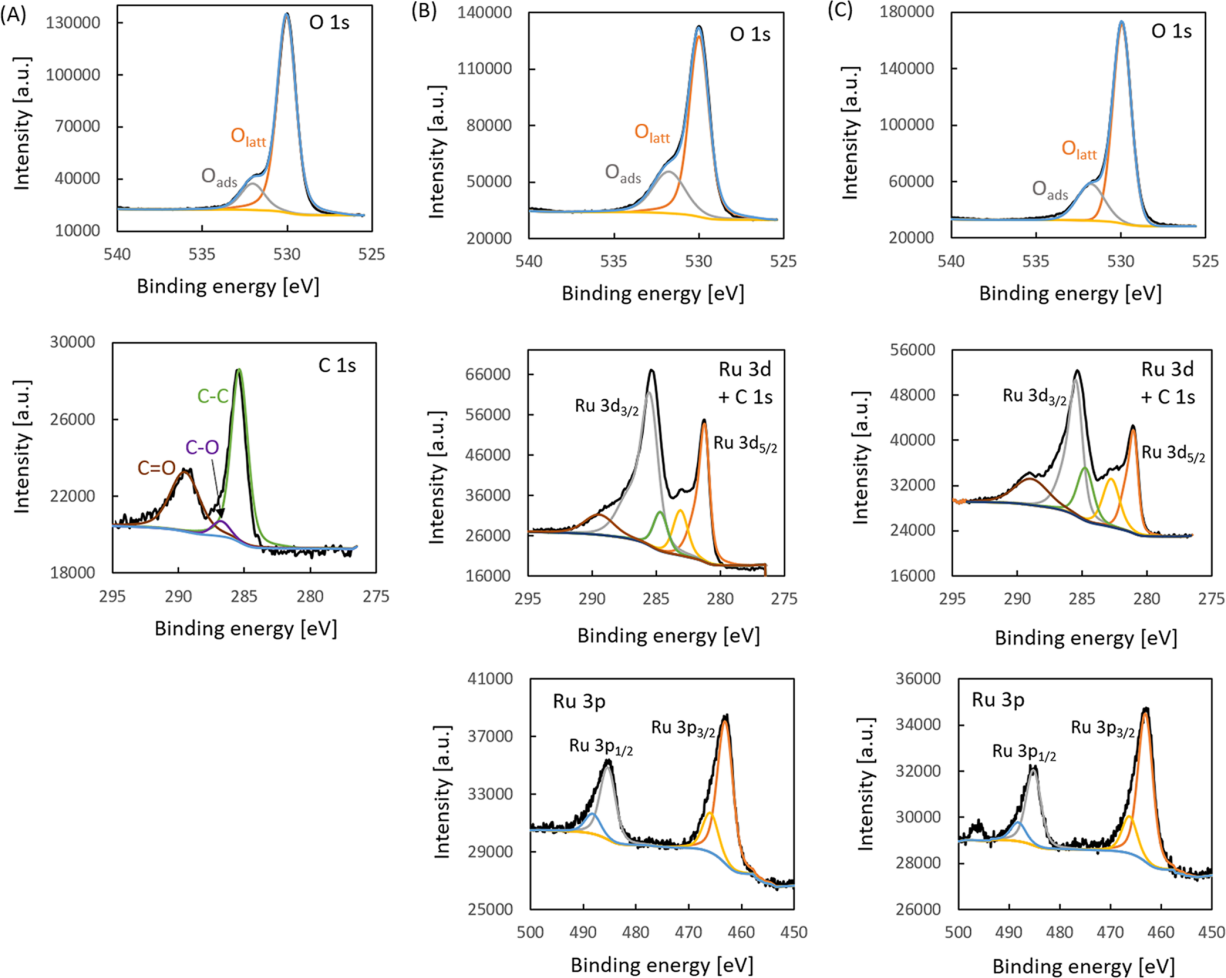
XPS results: fitted peaks of all detailed regions for (A) Na^+^/CZ, (B) RuO_2_/CZ, and (C) RuO_2_ + Na^+^/CZ.

The peaks in the detailed regions for Na^+^/CZ, RuO_2_/CZ, and RuO_2_ + Na^+^/CZ were fitted after the main O 1s component was shifted to 530.0 eV. The fitted peaks in the O 1s (Figure 4 row 1), Ru 3d + C 1s (Figure 4 row 2),and Ru 3p (Figure 4 row 3) regions are compiled in Figure 4. The RuO_2_/CZ sample does not contain sodium, whereas the other two have the same amount of sodium (0.3 atom %), and their Na 1s peaks have been fitted with a single component with a similar full width at half-maximum (fwhm; Figure S3). The O 1s regions (Figure 4 row 1) of Ru/CZ, Ru + Na^+^/CZ, and Na^+^/CZ systems can be fitted with two components. There is no change in the position of fitted peaks in the Ru 3p region (Figure 4 row 3), which is typically fitted with two doublets, the latter most likely a satellite. Hence, the Ru 3p region does not indicate a change in the chemical environment of ruthenium upon doping with sodium. The shape of the peaks in the 275–295 eV region in the two samples that contain ruthenium is typical for ruthenium oxide as reported by Morgan,^38^ Rochefort et al.,^39^ and Näslund et al.,^40^ as well as by Kaga et al.^41^ (Figure 4 row 2). The fitting of this region is difficult considering the fact that the Ru 3d signals overlap with C 1s peaks and consist of highly asymmetrical peaks with full width at half-maximum of the Ru 3d_3/2_ component almost twice that of the Ru 3d_5/2_ component. This is due to the Coster–Kronig broadening resulting from the filling of the M shell electron core hole with an electron from a higher subshell and hence a release of energy by an N-shell Auger electron.^41,42^ It is usually accepted that there are either two doublets: a low- and a high-energy doublet, or that there is one doublet and the two peaks at the higher BE values are satellites. The two doublets are attributed to two final states, which arise from localized d-level screening. In both cases, the C 1s peak is located between Ru 3d_5/2_ and Ru 3d_3/2_ peaks. Additionally, in the Na^+^/CZ sample, a pronounced carbonate peak is seen at the binding energy of 289.3 eV. This peak is also slightly larger in the case of the RuO_2_+Na^+^/CZ catalyst than that for RuO_2_/CZ, which is understandable, considering the fact that CO_3_^2–^ was the original counterion for the Na^+^ ions in the salt deposited onto the catalyst.

**Figure 5:**
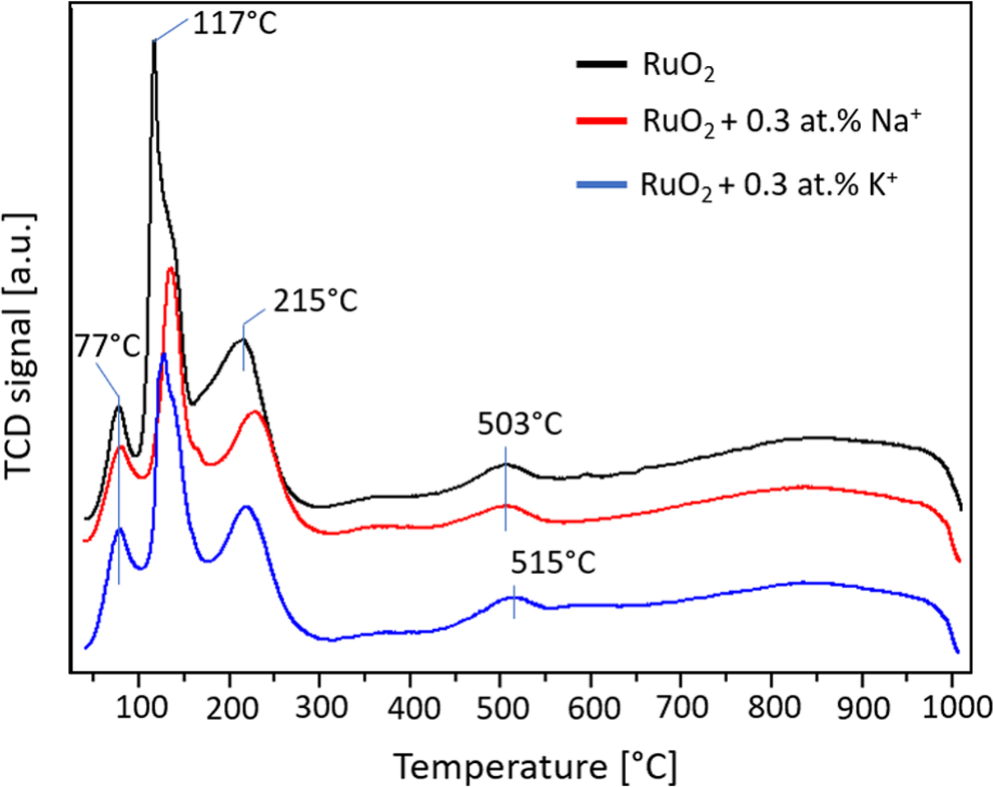
Temperature-programmed reduction curves of RuO_2_/CZ, RuO_2_ + Na^+^/CZ, and RuO_2_+ K^+^/CZ.

The shape of the TPR curve is not affected by deposition of alkali-metal ions, and there are three low-temperature reduction peaks, which can be attributed to the ruthenium species on the surface of the catalyst (Figure 5). It is likely that around 500 °C, the reduction of the surface of the support occurs, while at a higher temperature, the bulk is reduced. The values associated with these steps are collected in Table 1. A three-step reduction of a ruthenium oxide system in a TPR curve has also been reported by Ananth et al.,^43^ and the three steps were attributed to the “reduction of tiny RuO_2_ particles to metallic Ru” (peak 1), and the reduction of RuO_3_ (peak 2) and RuO_2_ (peak 3), respectively. This is highly unlikely because all of the results from methods that determine the elemental and phase composition point to RuO_2_ as being the predominant form of ruthenium in the catalyst, so the individual reduction peaks are probably caused by different particle sizes of RuO_2_ particles. The analysis of TPR curves reported by other research groups reveals that many factors, such as the shape and size of RuO_2_ particles, as well as their interactions with the support impact the number of peaks and the temperatures of the maximum rate of the reduction in TPR peaks.^43−45^ In our studies, the lack of a change in the shape of the TPR curves confirms that deposition of the alkali-metal ions did not influence these parameters, which is in line with the previously presented results (XRD, SEM-EDX).

The first reduction signal is very similar in both size and shape for all three samples, i.e., 77, 80, and 78 °C for RuO_2_/CZ, RuO_2_ + Na^+^/CZ, and RuO_2_ + K^+^/CZ, respectively. It is noteworthy that the presence of Na^+^ ions on the surface substantially decreases the amount of oxygen consumed in the second and third TPR peaks (Table 1). The quantity of hydrogen consumed drops from 7.0 to 4.1 and from 6.0 to 3.5 cm^3^/g, i.e., approximately 58.4 ± 0.2% of the original value in both cases. For potassium, the decrease is much lower and the values did not decrease by the same amount (14 and 25% from the original value). The presence of sodium ions has the most pronounced impact on the second reduction peak, whereas K^+^ influences the peak slightly above 500 °C, which is attributed to residual reduction of the support. This indicates that the sodium ions interact more with ruthenium oxide, whereas potassium ions interact more with the support. Since the theoretical hydrogen consumption required for the reduction of 1.8 wt % of ruthenium (if present entirely as Ru^4+^, namely, RuO_2_) to the metallic state is equal to 8.71 cm^3^ H_2_ /g, therefore, on the basis of the values listed in Table 1, we can surmise that, at least for RuO_2_/CZ and RuO_2_ + K^+^/CZ, the first three peaks account also for the reduction of some part of the CZ support. In the case of RuO_2_ + Na^+^/CZ, since the experimental hydrogen uptake value is coincident with the theoretical one and assuming all of the ruthenium is RuO_2_, we have to conclude that RuO_2_ particles are not in good contact with the support. Therefore, once metallic Ru is formed, it is not able to promote the reduction of some part of the support below 500 °C.

Apart from these standard characterization techniques, three additional techniques that pertain to the studied reactions were applied. These techniques aimed to point to the fact that the response of the catalyst rather than just its composition is important for activity. In other words, the presence of particular species is not the same as the ease of product desorption (CO_2_ desorption studies), extraction of ions from the surface (ToF SIMS), or the change of the support lattice associated with vacancy formation (dynamic in situ XRD studies in variable atmosphere).

**Figure 6:**
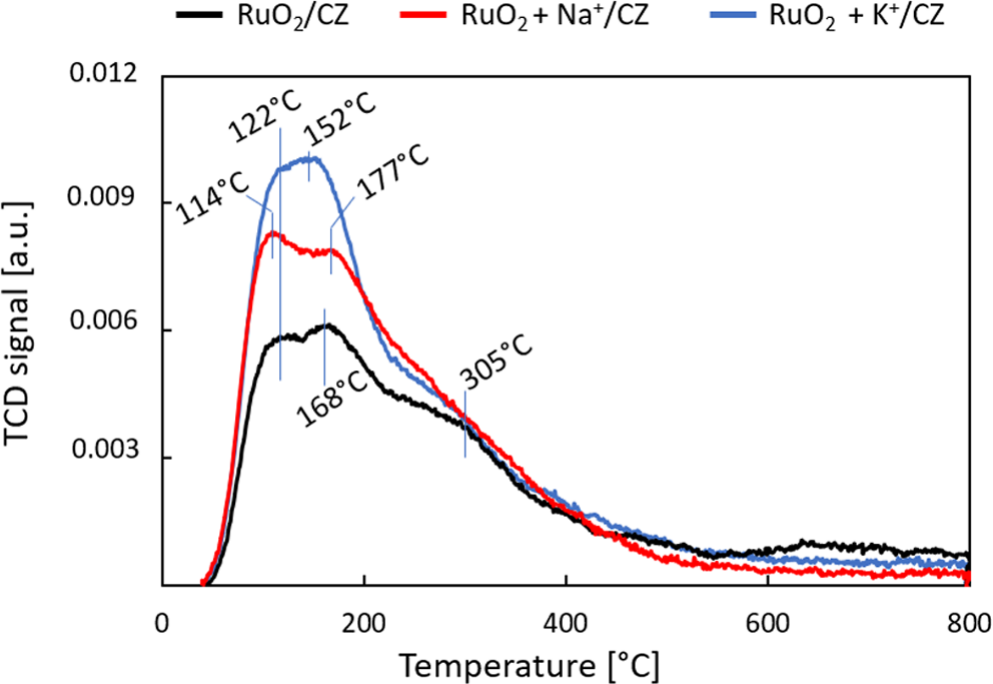
Results of the temperature-programmed desorption of carbon dioxide.

The influence of potassium and sodium ions on the surface of the novel ruthenium catalyst was further investigated by using CO_2_ temperature-programmed desorption (CO_2_-TPD). The results are listed in Figure 6. The CO_2_-TPD curves reveal that both alkali-doped systems exhibit more weak basic sites than the undoped catalyst since the amount of CO_2_ desorbed at temperatures below 200 °C decreases in the order RuO_2_/CZ > RuO_2_ + Na^+^/CZ > RuO_2_ + K^+^/CZ. The values are provided in Table 1. Upon the addition of Na^+^, there is a 25% increase in the amount of CO_2_ desorbed by the system and for the K^+^-doped sample an increase of 37% of the original value has been observed. Otherwise, the shapes of the desorption curves are very similar (Figure 6). In the case of the undoped system, there are two low-temperature peaks in the desorption curve, namely, at 122 and 168 °C, which are followed by a pronounced shoulder, indicating desorption of CO_2_ from medium-strength basic sites, at 305 °C.

**Figure 7:**
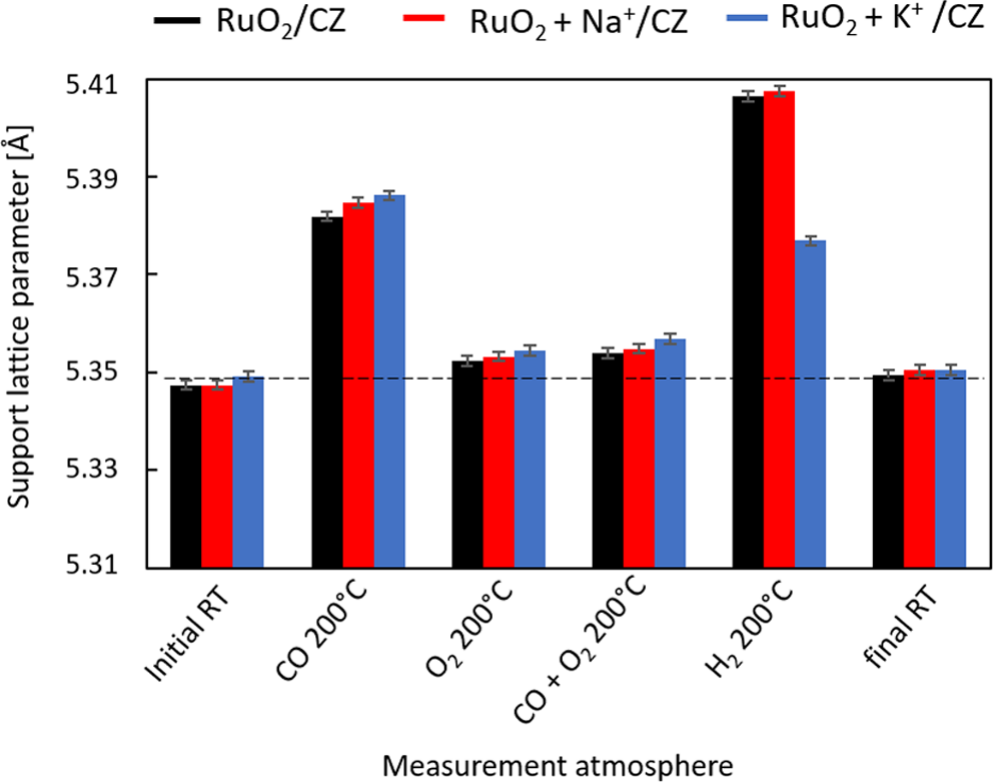
In situ XRD results: lattice parameter of the support in selected atmospheres and the CO oxidation reaction stream.

In situ XRD measurements during CO oxidation in a model stream were performed to understand how the presence of potassium and sodium ions influences the interaction of the catalyst surface with various stream components. The results in Figure 7 indicate a significant change in the lattice parameter of the support upon exposure to a CO stream at 200 °C. This difference is much larger than the temperature expansion of the unit cell and should therefore be attributed to the formation of Ce^3+^ ions in the presence of CO in the stream. It can be seen that the lattice parameter is slightly larger for the sodium-doped sample than for the undoped sample, and that in the potassium-doped sample it is even somewhat larger. This may indicate that the presence of alkali-metal ions causes an increased adsorption of CO onto the surface of the support.

The lattice parameter of the support in a stream of oxygen is much smaller than that in CO at the same temperature, and a similar value is noted when CO is added to the stream along with oxygen (Figure 7). In both cases, the trend is RuO_2_/CZ > RuO_2_+Na^+^/CZ > RuO_2_+K^+^/CZ. In contrast, when the catalyst is subjected to a hydrogen stream, the increase in the lattice parameter of the support reveals a pronounced difference in the reducibility of the support with and without potassium. It is noteworthy that the behavior of the sodium-doped sample does not resemble that of the potassium-doped sample. On the contrary, the value was practically the same as that of the undoped sample. This could explain the difference between the discrepancies in the activity trends observed for CO oxidation in the model and complex streams (Figure 7). After the last flushing with helium and decrease of temperature back to room temperature, the lattice parameter of the solid oxide returns to values that are very similar to the initial ones, which indicates that no irreversible changes have occurred during the in situ tests.

In this study, time-of-flight secondary ion mass spectrometry was used to probe the surface of the catalysts to determine how the doping of the catalyst with sodium and potassium ions influences the ease of extraction of oxygen from the surface. The advantage of this method is the high lateral resolution and precision with which one can select parts of the surface that are to be analyzed. In the literature, it has been shown that ToF SIMS can determine critical information about catalyst surfaces for the selective oxidation of isobutane,^46^ Fischer–Tropsch synthesis,^47^ ammonia synthesis,^48^ as well as catalytic systems for other reactions.^48,49^ A study on metallic catalysts supported on titania and alumina has been reported by J. Grams.^50^ However, these studies have not gained widespread application due to the main drawback of the method, which is the matrix effect, which, in short, means that the quantification of an element or species depends on its chemical environment. In other words, if two catalysts have the same active phase present in the same surface concentration on different supports, they will most likely give different ratios of fragments due to differences in the interaction of the active phase with different supports. In this study, however, the matrix effect is used as an advantage. Since ruthenium oxide was deposited onto one type of support and the catalysts differ only in the modification with alkali-metal carbonates, ToF SIMS can be a way to differentiate between the catalysts in terms of the ease of oxygen extraction from their surface, which depends on the chemical environment of oxygen.

The time-of-flight secondary ion mass spectrometry studies are carried out in both negative and positive ion mode. The resulting spectra show the relative abundance of fragments, which are placed on an *x*-axis with a mass-to-charge ratio. The fragments are often not the same as those found in the solid due to lack of stabilization by the lattice and hence in the case of oxides, typically the *m*/*z* = 16 ion, associated with a single negatively charged oxygen anion, O^–^, is the most abundant species. The stability of this ion outside of an oxide lattice was discussed by Adler.^51^

**Figure 8:**
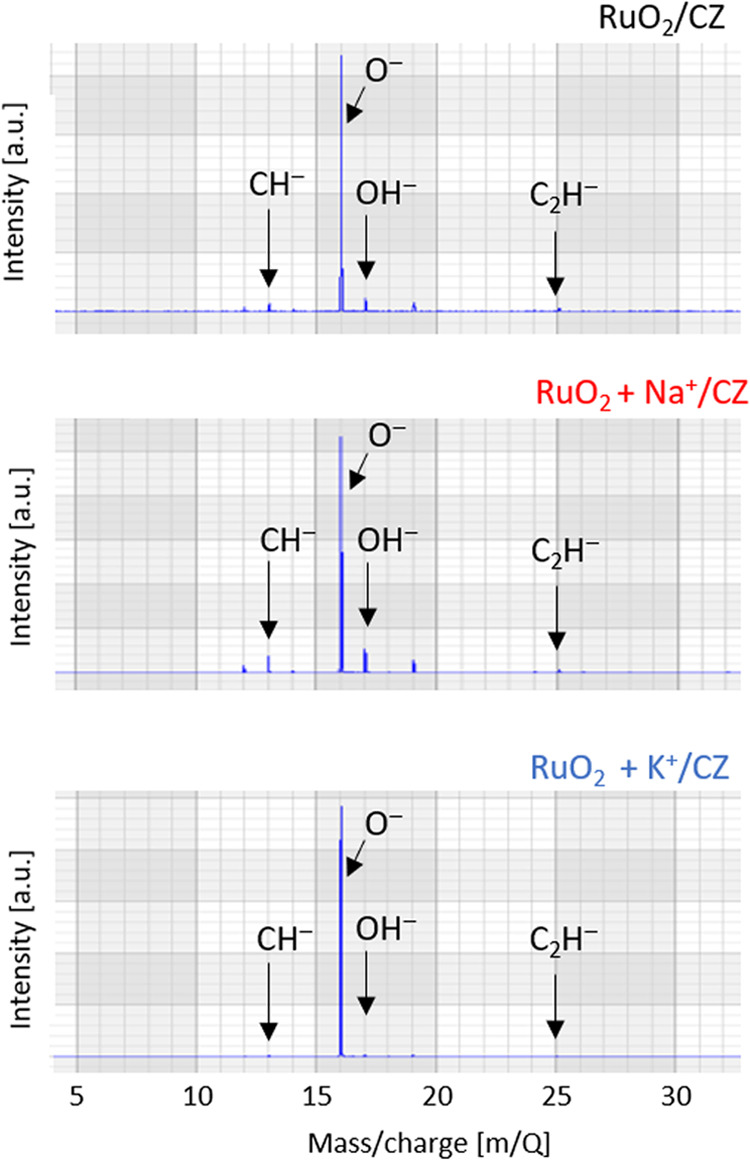
Time-of-flight secondary mass ion spectrometry measurement results: negative spectra obtained for RuO_2_/CZ, RuO_2_ + K^+^/CZ, and RuO_2_ + Na^+^/CZ.

Summing up this part of the studies, the properties of the catalysts determined by typical characterization techniques show that the presence of Na^+^ and K^+^ ions led to changes in the same direction of the studied parameters, e.g., XPS shows a decrease in the relative amount of adsorbed oxygen species upon doping, and CO_2_-TPD indicates increased basicity of the surface, ToF SIMS spectra revealed a significant differentiation of the oxygen ions emitted by the two alkali-doped surfaces, namely, the relative O^–^ and OH^–^ abundances in the spectra. These relative abundances differ from those observed using XPS due to the nature of the measurement. In XPS, the photoelectrons that are emitted from the oxygen atoms are detected, whereas in the case of ToF SIMS, secondary ions are emitted from the surface by striking it with primary ions. Hence, the relative intensities of the ions, as determined by ToF SIMS, correspond to the ease with which they are extracted from the surface. This is a key parameter for the catalytic behavior of the studied systems. The peak table was created with the four fragments shown in the negative ion spectra collected in Figure 8, i.e., the *m*/*z* signals 13 (CH^–^), 16 (O^–^), 17 (OH^–^), and 25 (C_2_H^–^), and the sum of their intensities was used to normalize the relative abundances of each species. Although the oxygen signal is the most intense of the four for all three catalysts, the relative abundances of O^–^ ions extracted from the surface are 98, 91, and 84 atom % for RuO_2_ + K^+^/CZ, RuO_2_/CZ, and RuO_2_ + Na^+^/CZ, respectively. In contrast, the OH^–^ ion was extracted with a far worse efficiency from the surface of the RuO_2_ + K^+^/CZ sample than the other two samples: for RuO_2_ + K^+^/CZ, RuO_2_/CZ, and RuO_2_ + Na^+^/CZ, the values were 1, 5, and 8 atom %.

**Figure 9:**
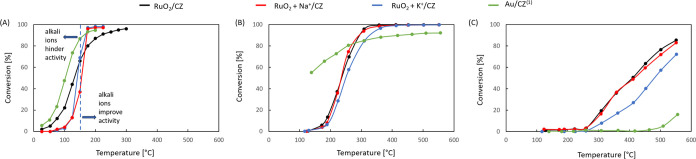
Activity measurement results: (A) CO oxidation in the model stream, and (B) CO oxidation as well as (C) propane oxidation in the complex stream. ^1)^ Data taken from ref (37).

High activity of a ruthenium catalyst in CO oxidation has been reported by other groups, e.g., in ref (43). However, in our studies, when compared with the results previously reported for the gold catalyst on the same support,^37^ it can be seen that the shape of the curves is the same, but the gold catalyst exhibits superior activity in the model stream (Table 2 and Figure 9A). The *T*_10_ and *T*_50_ values were collected in Table 2. For the undoped RuO_2_ and Au catalysts, the *T*_10_ temperatures are 97 and 69 °C, respectively. At *T*_50_, the gold catalyst continues to be better.

**Table 2:** Activity Measurement Results

	RuO_2_	Au	RuO_2_ + 0.3% Na^+^	RuO_2_ + 0.3% K^+^
Model Stream CO Oxidation				
*T*_10_ [°C]	97	69	117	117
*T*_50_ [°C]	130	102	154	140
Complex Stream CO Oxidation				
*T*_10_ [°C]	182	<115^a^	190	197
*T*_50_ [°C]	233	<115^a^	235	248
Complex Stream C_3_H_8_ Combustion				
*T*_10_ [°C]	282	533	289	322
*T*_50_ [°C]	391	>550^a^	393	460

a Tests were carried out in the temperature range of 115–550 °C.

The addition of 0.3 atom % sodium ions or 0.3 atom % potassium ions onto the surface of the ruthenium catalyst leads to two distinct regions of activity (Figure 9A): lower activity of the doped systems at lower temperatures and an increased activity at temperatures above 150 °C. Hence, the shape of the curves is different. An improvement of activity of a ruthenium catalyst upon alkali ion addition has been reported in the case of a Ru/AC catalyst for acetylene hydrochlorination with the optimum amount being approximately 0.5%.^52^ This effect was attributed to improved dispersion of Ru particles as well as inhibition of coke deposition. In the case of our study, deposition of alkali carbonates was performed after ruthenium hydroxide had been precipitated and calcined, which means that the improvement of the activity cannot be attributed to improved dispersion. However, the latter reason cannot be excluded for the improvement. This is in line with the fact that no such improvement is seen in the complex stream, where more oxygen and oxygen-containing species are available (Figure 9B). Although at T_10_ in the complex stream, the two alkali-doped catalysts exhibit slightly lower activity than the undoped ruthenium system (Table 2); at higher conversions, RuO_2_/CZ and RuO_2_+Na^+^/CZ are equally active (Figure 9B).

The inhibition of coke deposition may be a valid cause for the improved activity in the model stream, but this was not the case for the gold catalyst, which suffered an activity loss upon doping with alkali ions.^37^ In the case of the ruthenium catalyst, the addition of alkali ions hinders the activity only at lower temperatures (see *T*_10_ values, Table 2), especially in the model stream, but around 150 °C, the curve shows a rapid increase instead of a slow approach to its maximum value (Figure 9A).

The activity tests in the complex stream (Figure 9B) showed that although the gold catalyst was also more active in CO oxidation at lower temperatures in the complex stream, ruthenium systems exhibited a higher activity at higher temperatures. Interestingly, under these conditions, the catalyst doped with sodium (part of the same batch tested in the model stream) showed the same activity in CO oxidation as the undoped one, whereas the potassium-doped catalyst was slightly less active than the two others. Nevertheless, a 100% conversion is reached at approximately 400 °C. This could suggest that the activity of the Au catalyst is limited by the availability of active sites, whereas the activity of the Ru catalysts could be diffusion-limited at high conversions below 400 °C. It should be emphasized that the interpretation is much more obvious for the gold catalyst, which is a supported metallic catalyst and the active phase is comprised only of Au atoms. In contrast, the active phase of the ruthenium catalyst is ruthenium oxide and hence the activity can be influenced by both the ruthenium atoms present in that phase, as well as the lattice oxygen atoms. The proximity of oxygen ions and CO molecules on the surface of the particles required for the reaction to occur can be influenced by more factors than in the case of the gold catalyst.

It is noteworthy that the novel catalyst proved to be substantially superior to the gold catalyst in propane oxidation in the same complex stream (Figure 9C). In this case, the potassium-doped sample was observed to be substantially less active than the other two, reaching 85, 83, and 72% at 550 °C for RuO_2_/CZ, RuO_2_ + Na^+^/CZ, and RuO_2_ + K^+^/CZ, respectively. Thus, in this reaction, the ruthenium-based systems showed to be far superior to the gold catalyst studied by us previously.^37^ The fact that the potassium-doped sample has the most CO_2_ adsorption sites may be the reason it was slightly worse in the case of CO oxidation in the complex stream and much worse in propane combustion. CO_2_ is not only present in the complex stream feed but is also the product of both CO oxidation and, in larger quantities, propane combustion. Based on the dynamic in situ XRD experiment results, it is also possible that the composition of the gas stream changed in a way that affected the state of the catalyst. The juxtaposition of these facts points to areas of interest for future investigation.

## Conclusions

A novel catalyst in which ruthenium dioxide with a bimodal distribution of its particles is supported on a solid solution with a ceria structure was tested in CO oxidation. The addition of alkali-metal ions (Na^+^ and K^+^) to the ruthenium catalyst was performed to cause a slight modification of the surface and determine if and how these ions impact the catalyst surface properties. Differences in the influence of the alkali ions were noted in the two streams, and hence, apart from standard characterization techniques, more advanced methods were applied to actively probe the surface. In the model stream, the performance of the ruthenium catalyst in CO oxidation at intermediate temperatures was improved by the addition of alkali ions, possibly due to the inhibition of coke formation. No such effect was observed in the complex stream, which contained a higher concentration of oxygen as well as other oxygen-containing species. In fact, in the complex steam, the influence of sodium was negligible and that of potassium was slightly negative. The unique character of the potassium-doped sample was seen in the time-of-flight secondary ion mass spectrometry results, which showed that a plausible reason for the observed discrepancy is the difference in the ease of extraction of OH^–^ ions vs O^–^ ions and (hence a different O^–^/OH^–^ ratio) from the surface of the RuO_2_ + K^+^/CZ catalyst. In situ XRD measurements indicated that the interaction of the potassium ion-doped systems differs in the interaction with hydrogen, while the CO_2_ temperature-programmed desorption measurements revealed that it had the most CO_2_ adsorption sites. If the formation of CO_2_ during CO oxidation could lead to a slightly lower activity of the K-doped system in comparison to that of the other two, the substantially negative effect of potassium doping on the activity of the system in propane combustion can be attributed to larger quantities of CO_2_ formed during the reaction.

## Supporting Information Available

Isothermal hysteresis loop of RuO_2_/CZ (Figure S1); compilation of raw XPS detailed spectra of the three catalysts: (A) Ru 3p, (B) Ce 3d, (C) Zr 3d, (D) Na 1s, and (E) overlay of O 1s regions (Figure S2); and fitted XPS Na 1s peaks for (A) Na^+^/CZ and (B) RuO_2_/CZ, and RuO_2_ + Na^+^/CZ (Figure S3) (PDF)

## References

[ref1] https://single-market-economy.ec.europa.eu/sectors/automotive-industry/legislation/motor-vehicles-trailers_en. (accessed: June 29, 2024).

[ref2] Robles-Lorite L.; Dorado-Vicente R.; Torres-Jiménez E.; Bombek G.; Lešnik L. Recent Advances in the Development of Automotive Catalytic Converters: A Systematic Review. Energies 2023, 16, 6425 10.3390/en16186425.

[ref3] Chalapathi K. S.; Rao T. V. Development of a Cost Effective Catalytic Converter for Diesel Automobiles. Int. J. Recent Technol. Eng. 2019, 8, 2889–2894. 10.35940/ijrte.C4823.098319.

[ref4] Martínez-Munuera J.; Serrano-Martínez V. M.; Giménez-Mañogil J.; Yeste M. P.; García-García A. Unraveling the nature of active sites onto copper/ceria-zirconia catalysts for low temperature CO oxidation. Catal. Today 2022, 384–386, 246–256. 10.1016/j.cattod.2021.03.026.

[ref5] Piumetti M.; Bensaid S.; Fino D.; Russo N. Nanostructured ceria-zirconia catalysts for CO oxidation: Study on surface properties and reactivity. Appl. Catal., B 2016, 197, 35–46. 10.1016/j.apcatb.2016.02.023.

[ref6] Sun Y.; Li C.; Djerdj I.; Khalid O.; Cop P.; Sann J.; Weber T.; Werner S.; Turke K.; Guo Y.; Smarsly B. M.; Over H. Oxygen Storage Capacity versus catalytic activity of Ceria-Zirconia solid solutions in the CO and HCl oxidation. Catal. Sci. Technol. 2019, 9, 2163–2172. 10.1039/C9CY00222G.

[ref7] Eid K.; Gamalab A.; Abdullah A. M. Graphitic carbon nitride-based nanostructures as emergent catalysts for carbon monoxide (CO) oxidation. Green Chem. 2023, 25, 1276–1310. 10.1039/D2GC02748H.

[ref8] Choi S.-I.; Young A.; Lee S. R.; Ma C.; Luo M.; Chi M.; Tsung C.-K.; Xia Y. Pd@Rh core–shell nanocrystals with well-defined facets and their enhanced catalytic performance towards CO oxidation. Nanoscale Horiz. 2019, 4, 1232–1238. 10.1039/C9NH00360F.

[ref9] Ngorot Kembo J. P.; Wang J.; Luo N.; Gao F.; Yi H.; Zhao S.; Zhou Y.; Tang X. A review of catalytic oxidation of carbon monoxide over different catalysts with an emphasis on hopcalite catalysts. New J. Chem. 2023, 47, 20222–20247. 10.1039/D3NJ03074A.

[ref10] Over H.; Kim Y. D.; Seitsonen A. P.; Wendt S.; Lundgren E.; Schmid M.; Varga P.; Morgante A.; Ertl G. Atomic-Scale Structure and Catalytic Reactivity of the RuO (110) Surface. Science 2000, 287, 1474–1476. 10.1126/science.287.5457.1474. 10688793

[ref11] Over H. Surface chemistry of ruthenium dioxide in heterogeneous catalysis and electrocatalysis: from fundamental to applied research. Chem. Rev. 2012, 112, 3356–3426. 10.1021/cr200247n. 22423981

[ref12] Öström H.; Öberg H.; Xin H.; LaRue J.; Beye M.; Dell’Angela M.; Gladh J.; Ng M. L.; Sellberg J. A.; Kaya S.; Sorgenfrei F.; Mercurio G.; Nordlund D.; Schlotter W. F.; Föhlisch A.; Wolf M.; Wurth W.; Persson M.; Nørskov J. K.; Abild-Pedersen F.; Ogasawara H.; Pettersson L. G. M.; Nilsson A.; et al. Probing the Transition State Region in Catalytic CO Oxidation on Ru. Science 2015, 347, 978–982. 10.1126/science.1261747. 25722407

[ref13] Timmer P.; Glatthaar L.; Weber T.; Over H. Identifying theActive Phase of RuO_2_ in the Catalytic CO Oxidation Reaction, Employing Operando CO Infrared Spectroscopy and Online Mass Spectrometry. Catalysts 2023, 13, 1178 10.3390/catal13081178.

[ref14] Gao F.; Goodman D. W. CO Oxidation over Ruthenium: Identification of the Catalytically Active Phases at near-Atmospheric Pressures. Phys. Chem. Chem. Phys. 2012, 14, 6688–6697. 10.1039/c2cp40121e. 22473306

[ref15] Hess F.; Sack C.; Langsdorf D.; Over H. Probing the Activity of Different Oxygen Species in the CO Oxidation over RuO2 (110) by Combining Transient Reflection-Absorption Infrared Spectroscopy with Kinetic Monte Carlo Simulations. ACS Catal. 2017, 7, 8420–8428. 10.1021/acscatal.7b02838.

[ref16] Patrzałek M.; Zasada A.; Kajetanowicz A.; Grela K. Tandem Olefin Metathesis/α-Ketohydroxylation Revisited. Catalysts 2021, 11, 719 10.3390/catal11060719.

[ref17] Kojima M.; Abdellatif M. M.; Nomura K. Synthesis of Semicrystalline Long Chain Aliphatic Polyesters by ADMET Copolymerization of Dianhydro-D-glucityl bis(undec-10-enoate) with 1,9-Decadiene and Tandem Hydrogenation. Catalysts 2021, 11, 1098 10.3390/catal11091098.

[ref18] Tai C.-C.; Pitts J.; Linehan J. C.; Main A. D.; Munshi P.; Jessop P. G. In Situ Formation of Ruthenium Catalysts for the Homogeneous Hydrogenation of Carbon Dioxide. Inorg. Chem. 2002, 41, 1606–1614. 10.1021/ic010866l. 11896731

[ref19] Zhai P.; Xia M.; Wu Y.; Zhang G.; Gao J.; Zhang B.; Cao S.; Zhang Y.; Li Z.; Fan Z.; Wang C.; Zhang X.; Miller J. T.; Sun L.; Hou J. Engineering single-atomic ruthenium catalytic sites on defective nickel-iron layered double hydroxide for overall water splitting. Nat. Commun. 2021, 12, 4587 10.1038/s41467-021-24828-9. 34321467 PMC8319438

[ref20] Chalupczok S.; Kurzweil P.; Hartmann H.; Schell C. The Redox Chemistry of Ruthenium Dioxide: A Cyclic Voltammetry Study—Review and Revision. Int. J. Electrochem. 2018, 2018, 1273768 10.1155/2018/1273768.

[ref21] Paulista L. O.; Albero J.; Martins R. J. E.; Boaventura R. A. R.; Vilar V. J. P.; Silva T. F. C. V.; García H. Turning Carbon Dioxide and Ethane into Ethanol by Solar-Driven Heterogeneous Photocatalysis over RuO_2_- and NiO-co-Doped SrTiO_3_. Catalysts 2021, 11, 461 10.3390/catal11040461.

[ref22] Truszkiewicz E.; Kowalczyk K.; Dębska A.; Wojda D.; Iwanek E.; Kępiński L.; Mierzwa B. Methanation of CO on Ru/graphitized-carbon catalysts: Effects of the preparation method and the carbon support structure. Int. J. Hydrogen Energy 2020, 45, 31985–31999. 10.1016/j.ijhydene.2020.08.235.

[ref23] Wang F.; He S.; Chen H.; Wang B.; Zheng L.; Wei M.; Evans D. G.; Duan X. Active Site Dependent Reaction Mechanism over Ru/CeO_2_ Catalyst toward CO_2_ Methanation. J. Am. Chem. Soc. 2016, 138, 6298–6305. 10.1021/jacs.6b02762. 27135417

[ref24] Ma H. Y.; Wang G. C. A First-Principles Study of the Mechanism and Site Requirements for CO_2_ Methanation over CeO_2_-Supported Ru Catalyst. J. Phys. Chem. C 2021, 125, 18161–18169. 10.1021/acs.jpcc.1c04231.

[ref25] Shin J. H.; Kim G. J.; Hong S. C. Reaction properties of ruthenium over Ru/TiO_2_ for selective catalytic oxidation of ammonia to nitrogen. Appl. Surf. Sci. 2020, 506, 144906 10.1016/j.apsusc.2019.144906.

[ref26] Feng J.; Liu L.; Zhang X.; Wang J.; Ju X.; Li R.; Guo J.; He T.; Chen P. Ru nanoparticles on Y_2_O_3_ with enhanced metal–support interactions for efficient ammonia synthesis. Catal. Sci. Technol. 2023, 13, 844–853. 10.1039/D2CY02035A.

[ref27] Feng J.; Liu L.; Ju X.; Wang M.; Zhang X.; Wang J.; Chen P. Sub-Nanometer Ru Clusters on Ceria Nanorods as Efficient Catalysts for Ammonia Synthesis under Mild Conditions. ACS Sustainable Chem. Eng. 2022, 10, 10181–10191. 10.1021/acssuschemeng.2c01635.

[ref28] Li J.; Liu Z.; Cullen D. A.; Hu W.; Huang J.; Yao L.; Peng Z.; Liao P.; Wang R. Distribution and Valence State of Ru Species on CeO_2_ Supports: Support Shape Effect and Its Influence on CO Oxidation. ACS Catal. 2019, 9, 11088–11103. 10.1021/acscatal.9b03113.

[ref29] Wang Y.; Wang R. Effects of chemical etching and reduction activation of CeO_2_ nanorods supported ruthenium catalysts on CO oxidation. J. Colloid Interface Sci. 2022, 613, 836–846. 10.1016/j.jcis.2022.01.062. 35091258

[ref30] Liu Z.; Lu Y.; Confer M. P.; Cui H.; Li J.; Li Y.; Wang Y.; Street S. C.; Wujcik E. K.; Wang R. Thermally Stable RuO_x_–CeO_2_ Nanofiber Catalysts for Low-Temperature CO Oxidation. ACS Appl. Nano Mater. 2020, 3, 8403–8413. 10.1021/acsanm.0c01815.

[ref31] Okrushko E.; Seminko V. V.; Maksimchuk I.; Bespalova I.; Malyukin Y. V. Formation of oxygen vacancies in ceria-zirconia nanocrystals studied by spectroscopic techniques. Funct. Mater. 2018, 25, 439–444. 10.15407/fm25.03.439.

[ref32] Aßmann J.; Crihan D.; Knapp M.; Lundgren E.; Loffler E.; Muhler M.; Narkhede V.; Over H.; Schmid M.; Varga P. Understanding the Structural Deactivation of Ruthenium Catalysts on an Atomic Scale under both Oxidizing and Reducing Conditions. Angew. Chem. Int. Ed. 2005, 44, 917–920. 10.1002/anie.200461805. 15624225

[ref33] Liu Z.-P.; Hu P.; Alavi A. Mechanism for the high reactivity of CO oxidation on a ruthenium–oxide. J. Chem. Phys. 2001, 114, 5956–5957. 10.1063/1.1353584.

[ref34] Macyk W.; Kisch H. Photoassisted Catalytic Oxidation of Carbon Monoxide at Room Temperature. Monatsh. Chem./Chem. Mon. 2007, 138, 935–940. 10.1007/s00706-007-0637-y.

[ref35] Sutton J. E.; Lorenzi J. M.; Krogel J. T.; Xiong Q.; Pannala S.; Matera S.; Savara A. Electrons to Reactors Multiscale Modeling: Catalytic CO Oxidation over RuO_2_. ACS Catal. 2018, 8, 5002–5016. 10.1021/acscatal.8b00713.

[ref36] Joo S. H.; Park J. Y.; Renzas J. R.; Butcher D. R.; Huang W.; Somorjai G. A. Size Effect of Ruthenium Nanoparticles in Catalytic Carbon Monoxide Oxidation. Nano Lett. 2010, 10, 2709–2713. 10.1021/nl101700j. 20568824

[ref37] Iwanek (nee Wilczkowska) E. M.; Liotta L. F.; Williams S.; Hu L.; Calilung L. F.; Pantaleo G.; Kaszkur Z.; Kirk D. W.; Gliński M. Application of Potassium Ion Deposition in Determining the Impact of Support Reducibility on Catalytic Activity of Au/Ceria-Zirconia Catalysts in CO Oxidation, NO Oxidation, and C_3_H_8_ Combustion. Catalysts 2020, 10, 688 10.3390/catal10060688.

[ref38] Morgan D. J. Resolving ruthenium: XPS studies of common ruthenium materials. Surf. Interface Anal. 2015, 47, 1072–1079. 10.1002/sia.5852.

[ref39] Rochefort D.; Dabo P.; Guay D.; Sherwood P. M. A. XPS investigations of thermally prepared RuO_2_ electrodes in reductive conditions. Electrochim. Acta 2003, 48, 4245–4252. 10.1016/S0013-4686(03)00611-X.

[ref40] Näslund L.; Ingason A. S.; Holmin S.; Rosén J. Formation of RuO(OH)_2_ on RuO_2_-Based Electrodes for Hydrogen Production. J. Phys. Chem. C 2014, 118, 15315–15323. 10.1021/jp503960q.

[ref41] Kaga Y.; Abe Y.; Yanagisawa H.; Kawamura M.; Sasaki K. Ru and RuO_2_ Thin Films by XPS. Surf. Sci. Spectra 1999, 6, 68–74. 10.1116/1.1247890.

[ref42] Mårtensson N.; Nyholm R. Electron Spectroscopic Determinations of M and N Core Hole Lifetimes for the Elements Nb—Te (Z = 41–52). Phys. Rev. B 1981, 24, 7121 10.1103/PhysRevB.24.7121.

[ref43] Ananth A.; Gregory D. H.; Mok Y. S. Characterization and Shape-Dependent Catalytic CO Oxidation Performance of Ruthenium Oxide Nanomaterials: Influence of Polymer Surfactant. Appl. Sci. 2015, 5, 344–358. 10.3390/app5030344.

[ref44] Shi J.; Hui F.; Yuan J.; Yu Q.; Mei S.; Zhang Q.; Li J.; Wang W.; Yang J.; Lu J. Ru-Ti Oxide Based Catalysts for HCl Oxidation: The Favorable Oxygen Species and Influence of Ce Additive. Catalysts 2019, 9, 108 10.3390/catal9020108.

[ref45] Feng J.; Liu L.; Ju X.; Wang J.; Zhang X.; He T.; Chen P. Highly Dispersed Ruthenium Nanoparticles on Y2O3 as Superior Catalyst for Ammonia Decomposition. ChemCatChem 2021, 13, 1552–1558. 10.1002/cctc.202001930.

[ref46] De Smet F.; Devillers M.; Poleunis C.; Bertrand P. Time-of-flight SIMS study of heterogeneous catalysts based on praseodymium and molybdenum oxides. J. Chem. Soc., Faraday Trans. 1998, 94, 941–947. 10.1039/a707883h.

[ref47] Grams J.; Ura A.; Kwapiński W. ToF-SIMS as a versatile tool to study the surface properties of silica supported cobalt catalyst for Fischer–Tropsch synthesis. Fuel 2014, 122, 301–309. 10.1016/j.fuel.2014.01.005.

[ref48] Rogowski J. TOF-SIMS study of morphology and chemical composition of wustite-based precursor and iron catalyst for ammonia synthesis. Appl. Surf. Sci. 2019, 469, 82–89. 10.1016/j.apsusc.2018.10.271.

[ref49] Weng L.-T. Advances in the surface characterization of heterogeneous catalysts using ToF-SIMS. Appl. Catal., A 2014, 474, 203–210. 10.1016/j.apcata.2013.08.029.

[ref50] Grams J. Surface studies of heterogeneous catalysts by time-of-flight secondary ion mass spectrometry. Eur. J. Mass Spectrom. 2010, 16, 453–461. 10.1255/ejms.1079. 20530830

[ref51] Adler D. Electronic Configuration of the O^2–^ Ion. J. Chem. Phys. 1970, 52, 4908–4909. 10.1063/1.1673733.

[ref52] Dai Y.; Xu X.; Zhu R.; Xie R.; Zhao C.; Yan Y.; Niu Q. The effect of alkali metals on the Ru/AC catalyst for acetylene hydrochlorination. Catal. Commun. 2021, 158, 106334 10.1016/j.catcom.2021.106334.

