# Supplementary material for: Active Probing of a RuO2/CZ Catalyst Surface as a Tool for Bridging the Gap Between CO Oxidation Catalytic Tests in a Model and Realistic Exhaust Gas Stream
Source: ACS Mater Au. 2024 Sep 24;4(6):643–53. doi: 10.1021/acsmaterialsau.4c00062 (PMC11565278; doi:10.1021/acsmaterialsau.4c00062)
Supplement: Supplementary file 1 — mg4c00062_si_001.pdf [file mg4c00062_si_001.pdf]

Supplementary material

Active Probing of a RuO_2/CZ Catalyst Surface as a Tool for Bridging the Gap Between CO Oxidation Catalytic Tests in a Model and Realistic Exhaust Gas Stream

Ewa M. Iwanek (nee Wilczkowska)[1,*], Leonarda Francesca Liotta [2], Giuseppe Pantaleo[2], Linje Hu[3], Shazam Williams[3], Donald W. Kirk[4] and Zbigniew Kaszkur[5]

[1] Faculty of Chemistry, Warsaw University of Technology, Noakowskiego 3, 00-664 Warsaw, Poland

[2] Istituto per lo Studio di Materiali Nanostrutturati (ISMN)-CNR, Palermo I-90146, Italy

[3] DCL International Inc., Concord, ON L4K 4T5, Canada

[4] Department of Chemical Engineering and Applied Chemistry, University of Toronto, 200 College St., M5S3E5, Toronto, ON, Canada

[5] Institute of Physical Chemistry, Polish Academy of Sciences, Kasprzaka 44/52, 01-224 Warsaw, Poland

[*] corresponding author: ewa.iwanek@pw.edu.pl

Number of pages: 3

Number of figures: 3

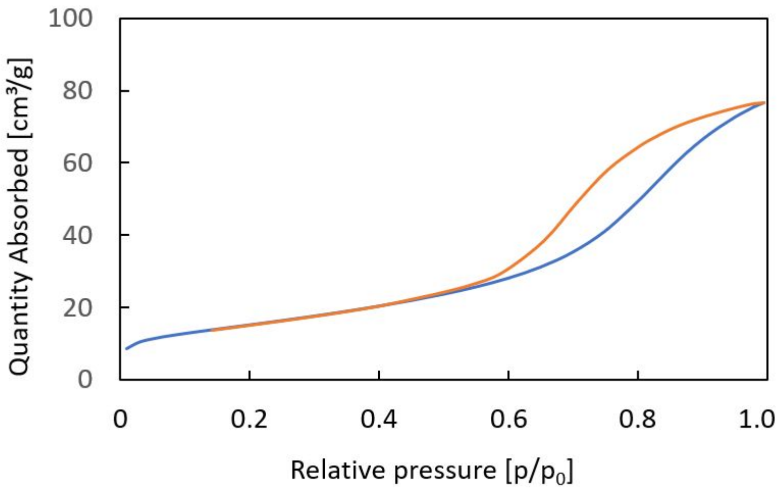

Figure S1. N_2 adsorption-desorption hysteresis loop of RuO_2/CZ.

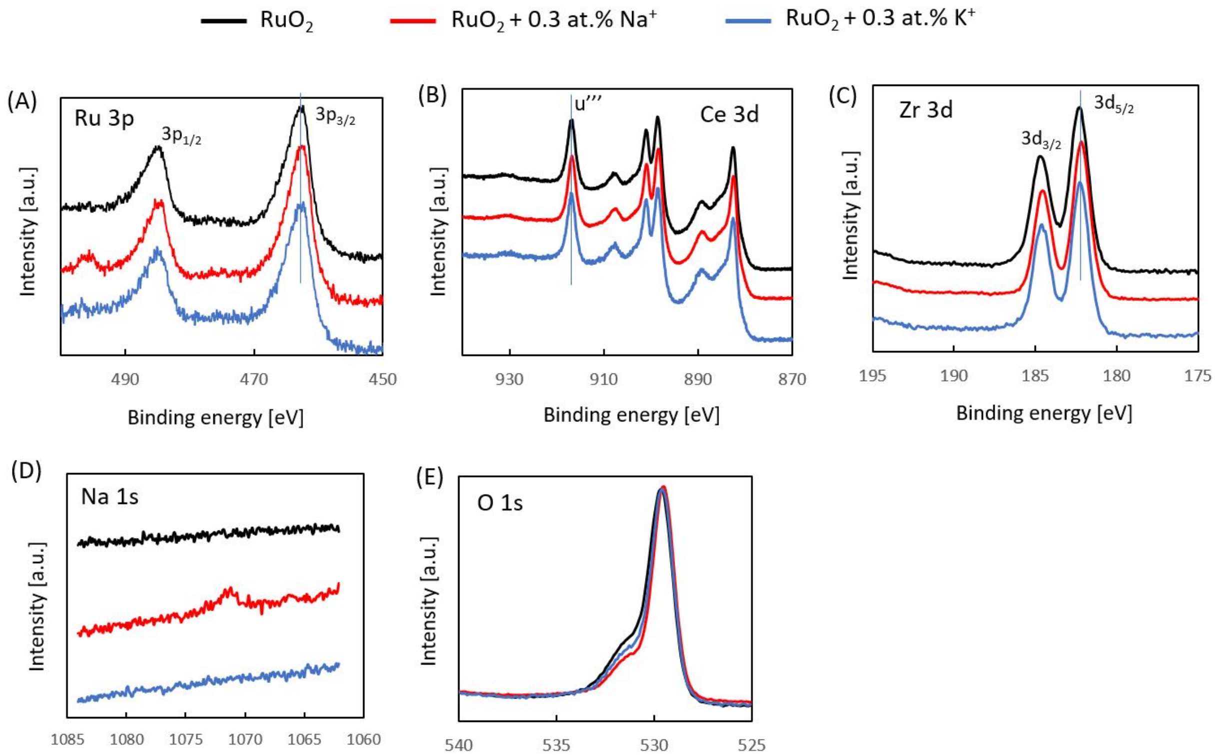

Figure S2. Compilation of raw XPS detailed spectra of the three catalysts: (A) Ru 3p, (B) Ce 3d, (C) Zr 3d, (D) Na 1s and (E) overlay of the O 1s peaks.

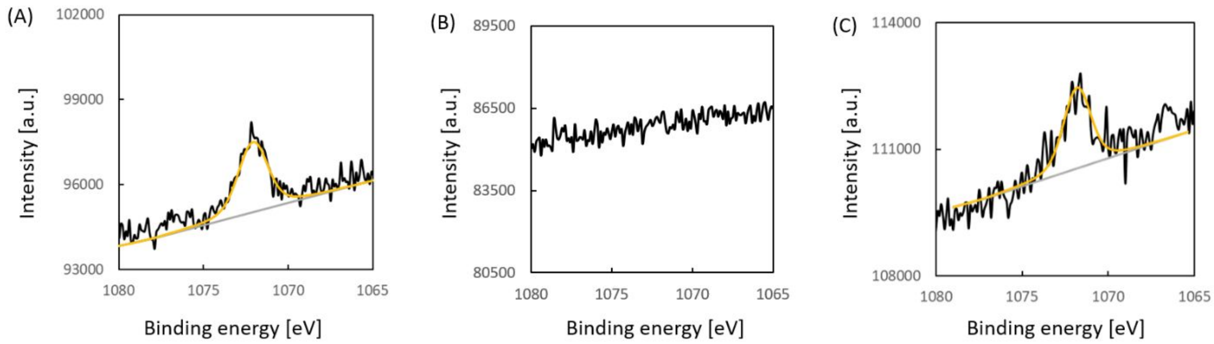

Figure S3. Fitted XPS Na 1s peaks for (A) Na^+/CZ, (B) RuO_2/CZ and RuO_2 + Na^+/CZ.
